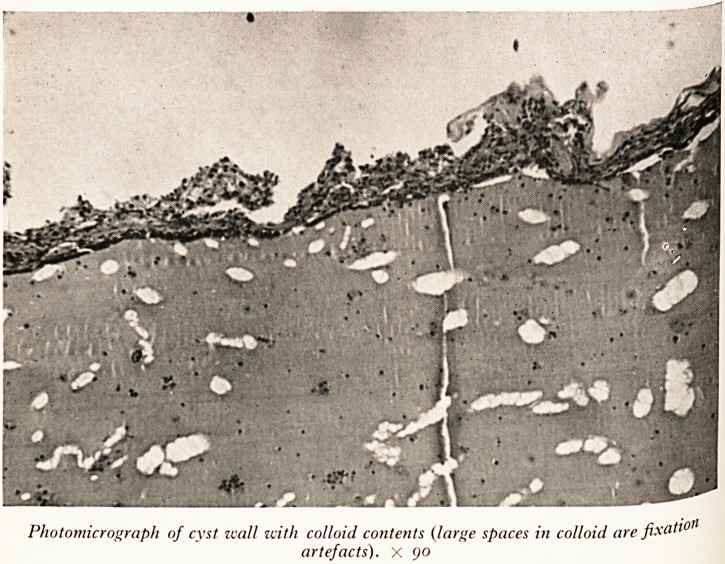# General Practice
*A paper read to Bristol Medico-Chirurgical Society on 14th April, 1954.


**Published:** 1954-10

**Authors:** G. F. Abercrombie

**Affiliations:** Chairman of Council, College of General Practitioners


### " GENERAL PRACTICE "*

BY

G. F. ABERCROMBIE, V.R.D., M.D.

(*Chairman of Council, College of General Practitioners*)

Mr. President, Ladies and Gentlemen,

I must first thank you very sincerely for a most flattering invitation to address this famous Society. I have undertaken it with some misgiving, for it is not easy to present a theme from general practice suited to your varied membership. I can only hope that some reflections on medical education may prove of interest to you, and I shall try to indicate what I have found of greatest value.

[Dr. Abercrombie then recapitulated one of those unfortunate cases, recently reported in the Press, in which a mistake in diagnosis combined with some lack of co-operation between hospital and practitioner had led to a tragedy. He continued:]

Here let me implore all general practitioners to follow their cases into hospital. Particularly is this desirable if the patient is a child. Never let us forget that when a patient goes into hospital she leaves outside her only real medical friend and adviser. A visit from such a one is an enormous comfort and encouragement, and if we feel that what we thought necessary has not been done, we can make the opportunity to seek out the man in charge and discuss the case with him.

Not only that, but we can explain to the patient what the hospital is doing. It is a very common complaint that " they tell you nothing in hospital ". The general practitioner then, sees the patients early, may see them as often as he wishes, may see them in their own homes, and should follow them into hospital to encourage and to explain.

Education, you will remember, has been defined as the casting of imaginary pearls before real swine, and as I pause to survey the long road so laboriously travelled, I find much force in that definition. Far away on the distant horizon are some low foot-hills, the preclinical subjects.

I may be wrong, but I am not conscious of being of inferior value to patients, because I now remember absolutely nothing of the anatomical structure of the skull of the dolphin, or of the reproductive arrangements of the earthworm; but I do very much regret that no one ever demonstrated to me the brain of the bird. It would have been, I think, very salutary for a medical student, at the stage when he had been immersed in the theory of man's evolution, to have been made to realize that, though apparently the most successful, the human brain is *not* Nature's last word on the subject. The avian brain is a later experiment and there may be others yet to come.

Of all that one was taught in those far-off days lamentably little now remains, but there are some scenes, not of detail, but of men, which have never been dimmed by the passing of years—scenes which, like beacon fires on mountain tops, have illumined some general principles of life-long value.

Having passed Anatomy and Physiology, three of us worked for some months as unqualified assistants to the Resident House Surgeon at Addenbrookes Hospital, Cambridge, and into our room one day strode Carless, already of course known to us by repute as part-author of the *Textbook of Surgery*, Rose and Carless, and I suppose in his day one of the most famous surgeons in England. He had come to operate on a soldier, whose sciatic nerve had been severed in mid-thigh, and he asked whether he might borrow our *Gray's Anatomy* for a moment to look up the relations of the

* A paper read to Bristol Medico-Chirurgical Society on 14th April, 1954.

sciatic nerve. I was never more astonished in my life—" Look up the relations of the sciatic nerve! " " Good God," I thought, " Doesn't he know them? " " Ah yes," he said, studying the diagram, " I see," and led the way to the theatre. What followed was to us intensely dramatic, an eighteen-inch incision down the back of the thigh, one other deft movement, and there it was. Now, if a master of surgery of that calibre was not too proud to revise his anatomy at a critical moment, surely we might follow his example, and certainly, from that day to this, I have never hesitated to look up anything.

Is it really necessary, I wonder, for every single one of us to make the tremendous intellectual effort to learn the whole topographical anatomy by heart? As those who aspire to be surgeons are nowadays compelled to return to the study of anatomy for the Primary Fellowship, would it not be reasonable to lessen the anatomical burden before qualification? And would not the same line of argument hold for biochemistry and physiology?

A little later, I was profoundly impressed by the physician, Drysdale of St. Bartholomew's in London, who in a very few words precisely defined the ideal doctor-patient relationship. " Gentlemen," he said, and he shook a finger at us, " you are not to suppose that you are sent into this world to order people's lives. You don't give orders; you give advice." No more valuable admonition could have been addressed to those who perhaps already were beginning to think they knew something, were somebody, and ought to be obeyed. " You don't give orders, you give advice." How exactly true that is. All our lives we are in effect saying to patients: " I advise you to stay in bed two more days; I advise you to have that wrist X-rayed; I advise you never to run for a bus "; and so on. There must not be any attempt at compulsion, and there ought not to be any attempt at argument. An argument between a doctor and his patient is generally undignified, seldom successful, and often leads to such loss of mutual trust and confidence that complete rupture follows. Much of the art of general practice lies in the management of one's fellowmen, and to secure the willing acceptance of unwelcome advice needs tact and skill.

What sort of answers, do you suppose, would be given by a student in his final examination, to questions such as these? It would be made clear of course, in a footnote, that no marks would be given for throwing up the case and running away.

1. You are called to a boy, two years old, and after careful consideration of the history of his illness and of the physical signs you are convinced that he has an intussusception. You are also convinced, from your previous knowledge of his parents, that at the mere mention of the word " operation " they will panic and dismiss you. How do you proceed?

2. In response to an urgent request received at 3.30 a.m. on a Boxing Day, you go to see a woman of thirty-eight, and although the way she rolls about in bed with abdominal pain is most unconvincing, you nevertheless form the opinion that it is an acute abdomen. You advise observation in a nursing-home, but this advice proves unacceptable to her and to her husband, who turn out to be Christian Scientists. What do you do now?

I had the good fortune to dress for and later to be House Surgeon to Sir Holburt Waring. What did he teach which has proved of value in the years of general practice? Certainly not surgery, for apart from a few well-worn formulae of which " Hernia, inguinal, right, indirect, epiplo enterocele, reducible, recurrent, non-strangulated " was a characteristic example, he appeared not to teach any scientific surgery at all. But Waring was a great man. He taught us to observe and he taught us to record, and any attempt at hedging was instantly pilloried. His most valuable contribution to me personally was his abhorrence of the word " slight ", which he would never allow to be used. " Slight oedema," " Slight dulness," " Slight tenderness "— these were out. Either the feet were swollen, or they were not; there was dulness over the base or there was not; the belly was tender or it was not; and we were absolutely forced to say and write one thing or the other. Not very long ago, I received from the radiological department of a hospital a six-line report, which contained the adjective " slight " three times. It was not of the slightest use.

Now this habit of making up one's mind and coming to a decision has been of very great help. Is this belly tender or is it not? If it is not, almost certainly all is well; but if it is, almost certainly it ought to be explored. I have practically given up the attempt at brilliant differential diagnoses in the acute abdomen, fascinating though such speculations are. I don't think the general practitioner is required to distinguish between, say, a ruptured ectopic gestation and a twisted ovarian cyst, or even between an acute appendix and a perforated peptic ulcer, but he *is* required to decide whether it is SAFE to leave such a patient in her own home throughout a long night. I entirely agree with the argument of a country practitioner that in such cases one ought to come to a firm decision on the spot at the first visit.

For a final illustration of this kind of instruction I must return to Cambridge to sit at the feet of Sir Clifford Albutt. " If I had my way," wrote this wise and humane physician, " no man, not even a pauper, should die of acute or obscure disease without a consultation." This plea is possibly less cogent now than when he first uttered it in 1889, but it is a constantly recurring situation of which inexperienced practitioners ought to be forewarned. Who is there among us who has not suddenly realized that the apparently trivial illness has unexpectedly taken a grave turn, or that, after two days in attendance, one must admit, if one is honest, that one really does not know what the diagnosis is? It has been said, too, that one cannot hope to diagnose a condition one has never seen and I assure you that I see, almost every month or two, something of which I have no previous experience, and which I am never likely to see again in private practice.

On too many occasions, I fear, the benefit of a second opinion, which Albutt so strongly advocates, is a good deal less than it should be. I sometimes think that those who offer these opinions in acute or obscure disease under-estimate the good sense of those who receive them. Robert Birley, now Headmaster of Eton, once remarked that, " if there is anything in the educational tradition of Western Europe, derived from the great teachers of Athens, the honest penetrating study of great literature or philosophy, or mathematics or science, should produce men of integrity, who can appreciate nonsense when they meet it." Now to give two examples: I may tell you what you will hardly credit, that I was offered the diagnosis of " streptococcal bronchorrhoea " for a man who was coughing up the contents of an abscess of the lung; and, for a woman who died of multiple new growths of the small intestine, " fatty diarrhoea with constipation ", was suggested. One of the subsidiary functions of the family doctor is to know when the consultant is bluffing. It is of course never easy to say " I don't know ", but it can be done and sometimes it ought to be done. I recently summoned a paediatrician to see a baby who was causing me some anxiety. After a thorough examination, he discussed the possibilities with me, and then turned to the mother and with disarming and quite admirable frankness, admitted that he could not make a diagnosis. " Your doctor has called me because he cannot put his finger on the exact spot of this trouble and *I am in the same position.* We ought to take him into a ward in the Children's Hospital, and investigate the urine, the cerebro-spinal fluid and so on."

Now that, if I may say so, is consultation at its best, and I have to add with very great regret that the National Health Service is killing it. In former days the consultant made his way largely by the good will and the good opinion of the general practitioner. But now he is independent, for his income is assured, and in consequence he is rapidly becoming an absolute autocrat. He is not to be found at the week-end, his night-work is done by his registrar and his houseman has instructions not to admit certain types of case from an Emergency Bed Service. This is not the occasion to discuss the allocation of hospital beds, but I cannot believe that conduct of that kind is a good example to students.

Some people will tell you that general practice is 90 per cent trivialities. I've never found it as dull as that. It depends entirely where your interest lies. If you are interested only in tonsils or hernias, leucocytes or spirochaetes, you will miss the heart of it, for general practice is concerned with men, women and children. If then you are interested in humanity, you will find it fascinating, though full of strange surprises, of which perhaps the student ought to be warned so that he may keep a straight face.

If, for instance, he should find an old lady sitting up in bed, and with a large pair of scissors cutting carefully along the lines of perforation in a roll of toilet paper, he must *not* say, "Madam, that material can easily be obtained in separate sheets, if you so desire." No, no, for what he witnesses is the newest branch of scientific medicine—Occupational Therapy.

Many years ago, I was asked to see at frequent intervals and at a considerable distance an old bankrupt dying of cancer and I was implored *not* to disclose to him the nature of his illness, of which, happily, he was ignorant. I did the best I could, but this assignment proved too difficult for me in my youth, and after two or three weeks the old man suddenly said, "Doctor, we're not making progress. I think I'd like a change." What could I say? Another man took my place, the patient died three days later. That kind of treatment is certainly hard, but one has to learn not to be hurt.

There is, of course, the other side of the picture. All successful doctors, as you know, sport a gold fountain-pen. Mine came to me as follows: For several days I missed the diagnosis of acute appendicitis in a stout Jewess, aged about fifty, in consequence of which the appendix perforated and an abscess formed which had to be drained; after that two sinuses persisted and a second operation had to be done about ten weeks later. Her illness in fact lasted nearly five months before her husband, in great gratitude, presented me with a remarkably fine gold pen, which was duly stolen from me by an Irish housemaid. However, the insurance company provided a poor substitute, which I have used ever since, and I still attend that old lady and we are very firm friends.

I think the young man who goes into general practice would appreciate a friendly warning that much disappointment lies ahead of him, and would welcome some advice as to how to make the best of it. Consider, here he is, eager, willing and sufficiently skilled to do quite a number of difficult things. He can remove an appendix, or a pair of tonsils; he can repair a hernia; he has probably had a good experience of the use of plaster of Paris in the treatment of fractures. He has learnt to put a hollow needle into the spinal theca and other tubes and cavities and perhaps into joints. I always like the old story of the two house physicians who had attempted to tap the pericardium, and considered that the fluid they withdrew was very deeply blood-stained. He can prepare with his own hand a tolerably good miscroscopic section; he may have mastered the technique of blood sugar and similar estimations, but when he gets into the ruck of general practice he will soon find it impossible to keep his hand in at everything. Suitable cases, so frequent in hospital, are uncommon in practice, and the care of equipment, always at hand in hospital and cleaned up and put away afterwards by someone else, takes more time than can be spared. And so he finds that, keen though he is, he has to let much of the varied and interesting work go away from him.

The best compensation is to choose some strictly limited field, which particularly appeals to him, put in half a day a week at it as a clinical assistant in hospital, and keep that going in his own practice.

[After discussing some of the problems of obstetrics Dr. Abercrombie turned to the subject of neurosis.]

This meant in those days—I am not sure what it means now—that if no adequate hypothesis could be found to fit the patient's symptoms no organic disease existed. I can see, in my mind's eye, an old man in my hospital whose prostate had been removed. After his operation he vomited over and over again. At first this was attributed to the anaesthetic, but after several days that diagnosis had to be abandoned and no other offering he was called "neurotic". Then he died. "How extraordinary," I thought, as I made my way to the post-mortem room, "that a man can actually die of a neurosis." And there, of course, as so often happens, the truth came out and the organic disease was demonstrated—in that case an obstruction by a distended oesophageal pouch.

That kind of thing will happen frequently unless one makes a rule, when confronted with persistent symptoms with or without physical signs, not to lose sight of the patient until a satisfactory explanation is forthcoming.

Let me illustrate this by recounting one of my own failures. On May 26th, in a certain year, I saw for the first time a man of thirty-eight who had been complaining of pain in his left shoulder for eight months. Novocain injections had given temporary relief and infra-red radiation would relieve him for half an hour. When severe, his pain spread to the arms and forearms, up the neck behind the ear and to the top of his head. It was not provoked by movement or exertion, but might come on when he was resting or during sleep. He had been passed for life assurance and I could find nothing the matter with him. I got him X-rayed. There was no evidence of any bone injury, disease or displacement in the shoulder, and no abnormal shadows in the soft tissues. There was no sign of any bony cervical rib and no evidence of any bony arthritic changes in the cervical spine. A masseuse helped him a great deal, but on July 6th—six weeks later—he still had two symptoms: (1) pain on the left side of the neck and up to the crown of the head when he lay down in bed at night, which might last half an hour or more before he fell asleep; and (2) a throbbing down the back of the left arm and into the palm of the hand via the inner side, coming on about 10 a.m. and lasting, if not relieved by veganin, several hours. Being completely stumped I took him to the best neurologist I know. This consultant confessed himself entirely unable to solve this problem in the absence of any physical sign to localize his symptoms, that is, any change in reflexes, any weakness, any wasting and so on. He might, he said, have a neuroma somewhere but he did not think lipiodol injection was really justified at the moment. Now for me, at any rate, this is the danger point. If I do not take a grip of myself, I am liable to begin to think he is a neurotic, and once I have labelled a patient " neurotic " I find it almost impossible to remain in sympathy with him, and to treat him as a reasonable being. No matter what he says, one still thinks, " Oh, he's only a neurotic ", and the number and gravity of mistakes which can be made in that way is appalling. However, they are not mistakes which can be overtaken and corrected. One has practically told the man there is nothing the matter with him and that it is all imagination. How is that going to be explained away when the truth is out?

Fortunately for my peace of mind I did not label this man a neurotic, for within two months he too was dead. A few days after the consultation with the neurologist, he took a little rowing exercise on the lake in Regent's Park, and a lump appeared in the middle of his right thigh towards the inner side. This gradually increased in size, and when he showed it to me on August 4th, it was an obivous aneurysm several inches in diameter. In hospital an X-ray of his chest revealed a large aneurysm of the aorta as well, which progressed rapidly. His Wassermann was positive, but massive anti-syphilitic treatment throughout failed. Finally the aneurysm in his thigh burst and he died the next day.

I have told you this long story to emphasize two points: first, if you cannot make a diagnosis, don't lose sight of the patient, keep him on the list, see him again and again, for something is sure to turn up sooner or later; and, second, don't label him neurotic and lose all interest and sympathy.

The recognition of early cases of cancer is immensely important, as you all know, and yet I find it one of the most difficult sides of our work. The public are easily frightened, which hampers us, for we are reluctant to cause unnecessary alarm and perhaps precipitate good people into anxiety states. Secondly, there is nearly always some apparently most cogent reason why this particular patient should not have cancer. Perhaps he has had this kind of flatulent dyspepsia for twenty years; perhaps his abdomen has been recently explored for some other condition, and we feel we know all about him. I can show you what I mean by two recent cases of carcinoma which illustrate the remarkable red herrings liable to be drawn across the path of the right diagnosis, and above all, demonstrate that when we take over a patient, we must never take over the previous diagnosis as well, until we have confirmed it for ourselves. No matter what other people have said, and no matter how eminent or distinguished they may be, it won't do to take their word for it. We must investigate for ourselves. There may be something else.

The first was a doctor, aged about sixty, formerly a psychiatrist, but now largely disabled by disseminated sclerosis, which he had had for many years. He was incontinent of urine and incidentally had been under the care of a surgeon, who had tunnelled his prostate and had been keeping him under regular observation. I was asked to go and see him about once a month and to let him talk. When one is young, one is inclined to think that that kind of visit is a complete waste of time and isn't really Medicine at all; but as one grows older one comes to realize that, to many people almost cut off from the rest of the world by some infirmity, the occasional visit by one of their remaining friends, in this case their doctor, is one of the few pleasures left to them. " I can't talk to Mr. So-and-So ", said a widow, referring to a consulting physician. " Why not," I asked, " he's a very charming man." " Yes, I know," she said, " but he always asks questions, and although I give him all the answers I don't feel I've *talked* to him."

One day my psychiatric friend said he was having a little trouble with his sphincter ani. Now, if there is disseminated sclerosis, it is very likely indeed that there will be trouble with the sphincters, and as I've told you, he had no bladder control at all. I naturally assumed that control of his bowel was affected by the same cause, but just to reassure him, I did actually put a finger into his rectum, and there to my horror, I felt a thing sticking into the lumen about the same size and shape as a cervix uteri, but very hard indeed. That man and his wife are very grateful, but I can confess to you, that we were all very fortunate.

About two years ago a colleague, who avoids obstetrics, asked me to attend a patient of his, whose second baby was due in a month or two. She was thirty-nine and after one child had had several miscarriages. This pregnancy therefore was probably her last chance of having another baby. She developed some piles towards the end of her time. She had had them before, she said, but had never bothered about them. During the first stage of labour the rectum was turned almost inside-out by pressure, and was pushed back from time to time to relieve the congestion. All went well and when she was fit to get up, I sent her back to her own doctor, told him the story, and said she really must have something done about her piles. He didn't examine her himself, but sent her straight on to a specialist, who reported that she had an annular carcinoma and advised an abdominoperineal. We had been so hypnotized by the confinement that this possibility had never entered our minds. Perhaps it was just as well, for what we should have done if we had discovered it in the last few weeks of pregnancy, Heaven only knows.

As to prognosis, I believe that that cannot be learned in hospital, and that only a long experience of sick people in general practice can give any knowledge of it. It is astonishing how long patients with a mortal illness can survive in comparative comfort, when in the care of skilled nurses and devoted relatives. The pinning or plating of a fractured femur in the elderly is now a commonplace and will generally save much suffering, but it is perhaps not so often realized that the wearing of a truss for many years adds greatly to the burden of old age. I do unhesitatingly advise a surgical opinion in such cases, even when the circumstances are obviously unfavourable.

I still find it extremely difficult to appreciate day-to-day or week-by-week changes in a patient's progress towards recovery or otherwise. When I began to practise and found a marked change or a new physical sign at a second examination, I could not help feeling this must be something which I had previously missed. This of course is most disconcerting and it has taken me many years to realize that the rate of change in signs can be almost unbelievably swift.

I wish that as a student, I had been initiated into some sort of Nosological Record, so that I might have kept as I went along lists of, say, the incidence and outcome of various types of malignant disease; the varieties of minor fractures, the success or otherwise of operations on the knee joint or the gall bladder or the prostate, and other interesting phenomena. I wish, too, that when I was a houseman, I could have seen something of the planning and organization which goes on in a big hospital. It would be no bad thing if housemen were taken by their chiefs, once in a while, to a meeting of the Council or Finance Committee or similar body. We all rise to positions of responsibility in one field or another and we ought to be introduced to this side of medical life, while we are still young. It would, I am sure, be a most stimulating and enlightening experience to listen to the formulation of the plan for a new wing or a new department, to watch an able chairman control an acrimonious discussion, or to hear a harrassed treasurer describe his financial resources.

In conclusion, Sir—and I must thank you for having been so patient with me—in conclusion I wish that, when I first entered general practice, there had been a College of General Practitioners. How eagerly one would have joined it! How much it might have accomplished in all these years!

Still, better late than never. We have made a start.

### PLATE XVIII

*Coronal section of brain showing cyst in 3rd ventricle*

*Photomicrograph of cyst wall with colloid contents (large spaces in colloid are fixation artefacts).* × 90

## Figures and Tables

**Figure f1:**
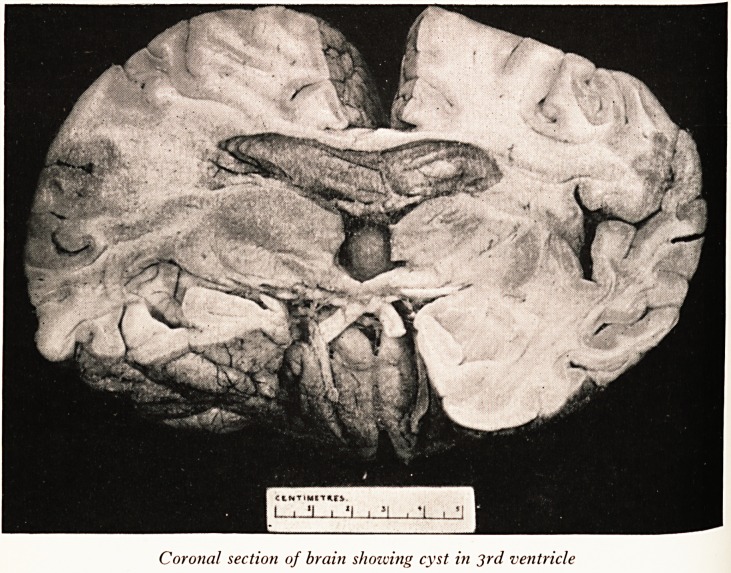


**Figure f2:**